# Structural connectivity centrality changes mark the path toward Alzheimer's disease

**DOI:** 10.1016/j.dadm.2018.12.004

**Published:** 2019-01-18

**Authors:** Luis R. Peraza, Antonio Díaz-Parra, Oliver Kennion, David Moratal, John-Paul Taylor, Marcus Kaiser, Roman Bauer

**Affiliations:** a Institute of Neuroscience, Newcastle University, Newcastle upon Tyne, United Kingdom; b Center for Biomaterials and Tissue Engineering, Universitat Politècnica de València, Valencia, Spain; c Interdisciplinary Computing and Complex Biosystems Research Group, School of Computing, Newcastle University, Newcastle upon Tyne, United Kingdom; d Institute of Genetic Medicine, Newcastle University, Newcastle upon Tyne, United Kingdom

**Keywords:** Alzheimer's disease, Diffusion MRI, Structural brain connectivity, Network centrality, Computational modeling, Machine learning

## Abstract

**Introduction:**

The pathophysiological process of Alzheimer's disease is thought to begin years before clinical decline, with evidence suggesting prion-like spreading processes of neurofibrillary tangles and amyloid plaques.

**Methods:**

Using diffusion magnetic resonance imaging data from the Alzheimer's Disease Neuroimaging Initiative database, we first identified relevant features for dementia diagnosis. We then created dynamic models with the Nathan Kline Institute-Rockland Sample database to estimate the earliest detectable stage associated with dementia in the simulated disease progression.

**Results:**

A classifier based on centrality measures provides informative predictions. Strength and closeness centralities are the most discriminative features, which are associated with the medial temporal lobe and subcortical regions, together with posterior and occipital brain regions. Our model simulations suggest that changes associated with dementia begin to manifest structurally at early stages.

**Discussion:**

Our analyses suggest that diffusion magnetic resonance imaging–based centrality measures can offer a tool for early disease detection before clinical dementia onset.

## 1 Introduction

Alzheimer's disease (AD) is the most common cause of neurodegeneration in old age. Out of the main risk factors for developing AD, age is the most influential one [1]. AD is characterized by continuous degradation involving a preclinical stage, followed by a phase of mild cognitive impairment, and ending with dementia in the strict sense [1], [2]. Experimental evidence indicates that pathophysiological alterations take place in the brain more than a decade before clinical decline [3], [4]. Therefore, the search for biomarkers for early diagnosis and development of disease-modifying treatments is an ongoing and challenging endeavor [5].

The presence of neurofibrillary tangles and amyloid plaques is the main pathological hallmark of AD [3], [6], [7]. One emerging hypothesis about the progression of AD posits that these toxic proteins originate in a particular area and propagate throughout neural fibers in a prion-like manner [8], [9], [10].

Network neuroscience has proven useful for understanding the impact of psychiatric and neurological disorders on brain-wide networks ([11], [12] and Supplementary Material). In particular, it has been shown that AD strongly disturbs connections between nodes [13], [14], especially those nodes occupying a central role in the network (hub nodes) [15], [16].

In this work, we investigated whether structural brain networks, as measured with diffusion magnetic resonance imaging (MRI), could serve as a tool in the diagnosis of prodromal dementia [15]. By using the Alzheimer's Disease Neuroimaging Initiative (ADNI) database, we first aimed to implement machine learning techniques to extract features that are altered in Alzheimer's dementia. We then incorporated data from the Nathan Kline Institute-Rockland Sample (NKI) database and created dynamical models of normal aging and AD to estimate the earliest detectable stage associated with dementia in the simulated disease progression.

## 2 Methods

**Fig. 1:**
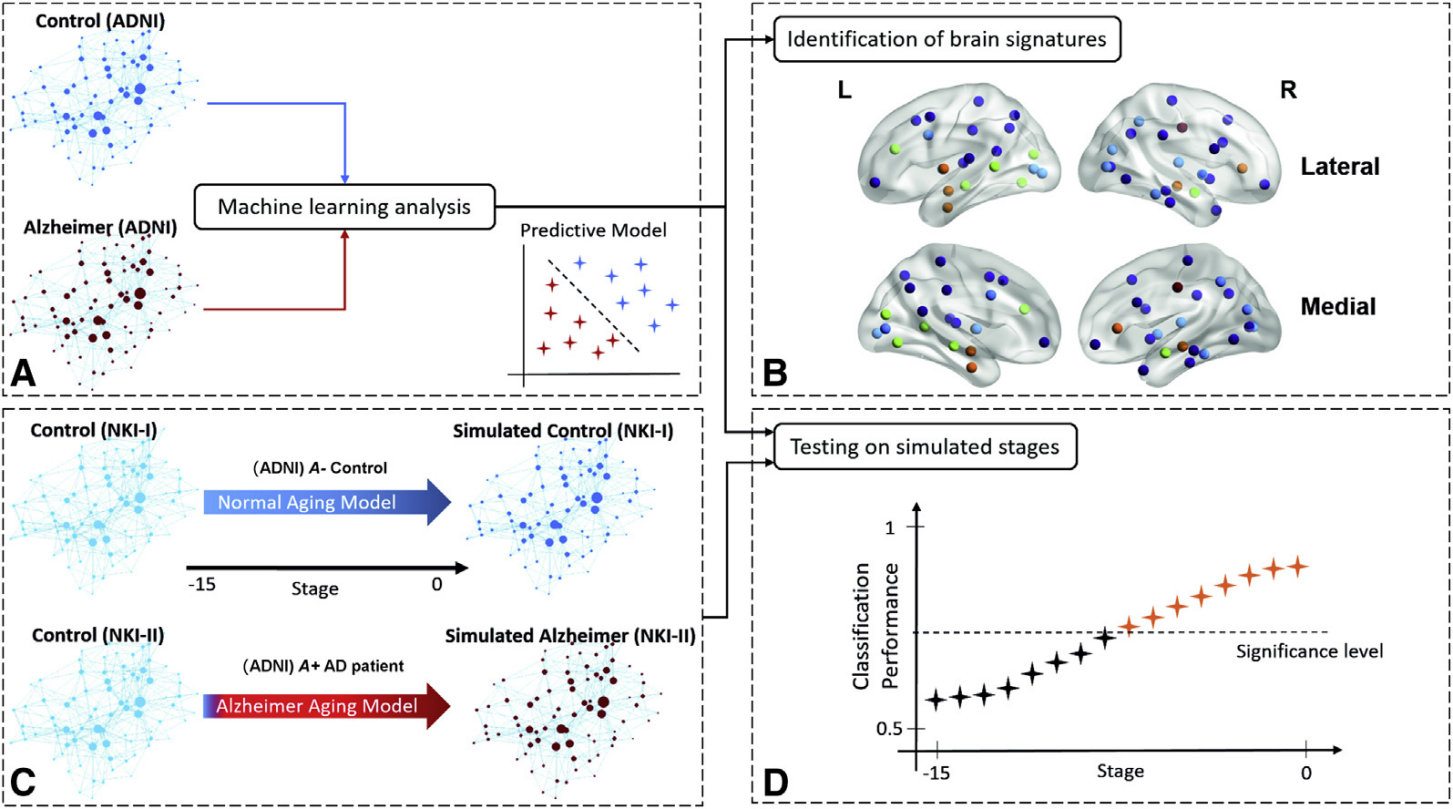
A basic schematic representation of the proposed approach. (A) In the first part of the work, we generated a predictive model based on measures of complex networks to classify between a cohort of patients with dementia and a cohort of matched healthy controls. (B) We then extracted informed brain signatures enabling diagnosis. (C) In the second part, we applied dynamic models to emulate changes in structural connectivity caused by normal aging on the one hand and degradation of structural connections caused by Alzheimer's disease on the other hand. (D) We finally explored when the relevant features and brain signatures associated with dementia begin to be evident in the simulated progression process. Significance level represents the minimum value from which classification performance is statistically significant. See section 2 for a deeper explanation about the proposed approach. Abbreviations: ADNI, Alzheimer's Disease Neuroimaging Initiative; NKI, Nathan Kline Institute-Rockland Sample.

Fig. 1 presents a general overview of the proposed approach, which is described in the following sections.

### 2.1 Participants and MRI acquisition protocol

We used two publicly available data sets widely used in neuroimaging research: the ADNI (http://adni.loni.usc.edu/) [17] and NKI (http://fcon_1000.projects.nitrc.org/indi/pro/nki.html) [18] databases (see also Supplementary Material). Here, we used 39 normal controls and 39 age- and sex-matched patients with AD and mild dementia, meeting National Institute of Neurological and Communicative Diseases and Stroke/Alzheimer's Disease and Related Disorders Association criteria for probable AD (http://adni.loni.usc.edu/wp-content/uploads/2008/07/adni2-procedures-manual.pdf). ADNI participants were then subdivided into amyloid-positive patients with AD (*A+*) and amyloid-negative healthy controls (*A−*) according to recommended standard uptake value ratio thresholds for the ADNI database [19] (Supplementary Material). These cohorts were used to generate a classifier predicting clinical AD dementia (Fig. 1A) and identify discriminative centrality metrics and brain regions allowing for such diagnosis (Fig. 1B). Afterward, we included 52 adults (26 men and 26 women) with age ranging from 45 to 81 years (mean ± standard deviation, 60.5 ± 10.41) from the NKI data set. These individuals are healthy with no presumed covert neuropathology (Supplementary Material).

We divided the NKI data set into two different groups: (1) NKI-I and (2) NKI-II. We then simulated an experiment over 15 years whereby one group experienced normal aging (NKI-I group), whereas another group of age- and sex-matched individuals developed AD over the same period (NKI-II group) (Fig. 1C). This second analysis allowed us to investigate how early structural network alterations associated with dementia take place in the simulated progression process (Fig. 1D). Table 1 summarizes the subject characteristics of the four groups considered in this work. For the ADNI data set, the education variable was marginally different between controls and patients (P=.047). There was a strong difference between controls and patients in the Mini-Mental State Examination score ($P=4.042\times \;10^{-13}$). MRI acquisition protocols are summarized in Supplementary Material.

**Table 1:** Subjects' information across groups

	ADNI			NKI		
	Control (n = 39)	Alzheimer's disease (mild dementia) (n = 39)	*P* value	NKI-I (n = 26)	NKI-II (n = 26)	*P* value
Age∗	74.19 ± 6.29	74.45 ± 8.57	.88	60.54 ± 11.01	60.46 ± 9.98	.979
Sex†	20 M/19 F	20 M/19 F	1	13 M/13 F	13 M/13 F	1
Education∗	16.38 ± 2.75	15.10 ± 2.86	.047	-	-	-
MMSE∗	28.67 ± 1.42	22.10 ± 4.47	4.042 × 10^−13^	-	-	-
SUVR∗	1.1 ± 0.14	1.38 ± 0.19	5.44 × 10^−10^	-	-	-
SUVR+/−	8+/29−	33+/4−	-	-	-	-

Abbreviations: ADNI, Alzheimer's Disease Neuroimaging Initiative; MMSE, Mini-Mental State Examination; NKI, Nathan Kline Institute-Rockland Sample; SD, standard deviation; SUVR, standard uptake value ratio; SUVR+/−, amyloid-positive or amyloid-negative confirmed participants.

NOTE. Education and MMSE variables were not available for NKI data set.

∗ Values represent mean ± SD. The group difference was evaluated using a two-sample *t*-test (two-tailed).

† Group difference evaluated using a *χ*^2^ test (two-tailed).

### 2.2 Preprocessing of MRI data and estimation of structural networks

The preprocessing pipeline to extract brain graphs from the NKI data set has been described elsewhere [20]. For the ADNI data set, a similar pipeline was applied, as described in Supplementary Material.

### 2.3 Mathematical models

#### 2.3.1 Alzheimer aging model

We implemented the susceptible-infected model [21], [22] to simulate the propagation of a disease factor as AD progresses. In this model, nodes can be in two possible states, infected or susceptible. Infected nodes are brain regions wherein the probability of the disease factor burden is greater than zero. By contrast, susceptible nodes are free of the disease factor but are susceptible to be infected from other nodes. In a network with *N* nodes, for any particular node i, the probability of being susceptible, $s_{i}$, and the probability of being infected, $x_{i}$, satisfy such that $s_{i}+x_{i}=1$. Thus, it is possible to express the rate of change of $x_{i}$ in the susceptible-infected model as [21]

(1) $$\frac{dx_{i}}{dt}=\alpha \left(1-x_{i}\right)\sum_{\begin{array}{l} j=1\\ j\neq i \end{array}}^{N}a_{ij}x_{j}\text{,}$$

where $a_{ij}$ is an element of the (binary) adjacency matrix, whereas the parameter alpha, α>0, controls the infection rate of the node j over the node i. As we made use of weighted networks, we replaced equation (1) with

(2) $$\frac{dx_{i}}{dt}=\alpha \left(1-x_{i}\right)\sum_{\begin{array}{l} j=1\\ j\neq i \end{array}}^{N}\frac{w_{ij}}{k_{j}}x_{j}$$

In equation (2), $k_{j}$ stands for the strength of node j, and the influence of the node j over the node i is now proportional to the weight $w_{ij}$. We incorporated another equation simulating AD-related degradation in connectivity. Particularly, the rate of change of $w_{ij}$ is given by

(3) $$\frac{dw_{ij}}{dt}=-\beta^{AD}w_{ij}\left(x_{i}+x_{j}\right)+\beta_{ij}^{N}\text{,}$$

where the parameter beta, $\beta^{AD}>0$, controls the influence of the aggregated disease factor present in both node i and node j on the number of streamlines connecting them. The term $\beta_{ij}^{N}$ represents the normal aging process (subsection 2.3.2) to reflect the fact that patients also age. Therefore, the Alzheimer aging model (equations (2) and (3)) relies on three unknown parameters: α, $\;\beta^{AD}$, and the seed region origin of the disease factor. By varying these parameters, one can simulate different disease trajectories.

#### 2.3.2 Normal aging model

We developed a mathematical model reflecting the process through which structural connections change due to normal aging. Connectome organization develops across the lifespan, with age and sex being two critical factors during this process [20], [23]. We created link-specific regression models to predict the number of streamlines from age- and gender-related effects. Inspired by Lim et al. [20], the number of streamlines between region i and region j, $w_{ij}$, can be expressed as a combination of *P* = 4 variables or predictors (age, gender, age gender, age^2^):

(4) $$w_{ij}=\beta_{0}^{ij}+\beta_{1}^{ij}age+\beta_{2}^{ij}gender+\beta_{3}^{ij}age\;gender+\beta_{4}^{ij}age^{2}$$

where gender = 1 for males and gender = −1 for females. In equation (4), we included an intercept term ($\beta_{0}^{ij}$), the linear effect of both age ($\beta_{1}^{ij}$) and gender ($\beta_{2}^{ij}$), the interaction between age and gender ($\beta_{3}^{ij}$), and the quadratic effect of age ($\beta_{4}^{ij}$). The coefficients from equation (4) were only estimated for those connections that all NKI participants had in common (126 connections), generating a minimum grid mask [24]. Thus, we had 52 data points over the age range of 45 to 81 years for each of the 126 common connections.

Upon deriving equation (4) with respect to the age effect, we obtained the following differential equation attempting to mimic the process for aging:

(5) $$\frac{dw_{ij}}{dt}=\beta_{ij}^{N}=\beta_{1}^{ij}+\beta_{3}^{ij}gender+2\beta_{4}^{ij}age$$

It has been shown that brain connectivity evolves in such a manner that core properties of structural networks (e.g., modular organization) remain stable during brain maturation and adulthood [20], [23], [25], suggesting that rules governing neural fiber changes over time are dependent on connections. In mathematical terms, it means that some predictors from equation (4) can be irrelevant to predict the number of streamlines. We, therefore, applied the model-selection algorithm named subset selection (see [26] and Supplementary Material) to exclude variables not related to the response $w_{ij}$. The outcome of this procedure was a set of link-specific regression models (equation (4)) and their corresponding differential equations (equation (5)), varying in complexity according to the number of included predictors. Details for the parameter fitting within the aging model can be found in the study by Lim et al. [20].

#### 2.3.3 Dynamical simulations

We applied the aforementioned normal aging model to the NKI-I group to create a cohort of simulated healthy controls evolving over 15 years. Similarly, we applied the Alzheimer aging model to the NKI-II group to reproduce the process of propagation of misfolded proteins and disruption of neural pathways throughout the same time window, creating a cohort of simulated patients with AD (see Fig. 1C and Supplementary Material).

We recorded the resulting simulated matrices in discrete points of a year and defined a set of 16 simulated stages for further analysis (Fig. 1D). From this point on, we will be using the term “stage” rather than “years” to account for the fact that patients can develop AD at different rates. Thus, stage 15 represents the initial stage in which both groups of individuals (i.e., NKI-I and NKI-II groups) are in a healthy condition. By contrast, stage 0 represents the disease stage in which neural degeneration compromises cognitive functions enough to meet criteria for dementia. To assess the extent to which stage 0 reflects this criterion, we compared the differences in node strength obtained at stage 0 between simulated controls and simulated patients, with the counterpart differences measured between ADNI *A−* controls and ADNI *A+* patients. Specifically, we selected the parameter set $\left(seed,\alpha ,\beta^{AD}\right)$, minimizing the cost function defined by the Euclidean distance between $k_{NKI}^{0}$ and $k_{ADNI}$. The term $k_{NKI}^{0}$ represents the size 82 vector incorporating differences in node strength measured between simulated controls and simulated patients at stage 0, and the term $k_{ADNI}$ stands for the size 82 vector containing the differences in node strength measured between ADNI *A−* controls and ADNI *A+* patients.

### 2.4 Feature extraction and machine learning analysis

We used the Brain Connectivity Toolbox v2017-15-01 (https://sites.google.com/site/bctnet/) [27] and computed centrality measures of strength, betweenness, closeness, eigenvector, and PageRank, producing 410 elements. Before computing the foregoing topological properties, the fraction of streamlines connecting each pair of regions was calculated [28]:

(6) $$w_{ij}^{\prime }=\frac{w_{ij}}{\sum_{k,l}w_{kl}}\text{,}$$

where *w*_*kl*_ represents the streamline counts connecting region k and region l.

Centrality metrics were scaled within subjects having zero mean and unit variance. Then, we applied machine learning techniques, as implemented in the Scikit-Learn Package v0.19.0 [29], to produce relevant features and brain patterns or signatures, classifying between clinically diagnosed ADNI controls and ADNI patients with dementia (Fig. 1A and B).

We used the random forest (RF) technique [26] with 1000 trees for feature selection [30], [31], with the ultimate goal of reducing model complexity and improving interpretability. This method returned a set of scores (feature importance) that were used to rank features for subsequent classification. The classification was performed using the support vector machine (SVM) technique [32]. We trained SVMs with a radial basis function kernel with *γ* = 1/*n*_*feat*_ (where *n*_*feat*_ denotes the number of features). The parameter *C* of radial SVMs was selected within the set (1,10,100) to maximize model performance. To estimate model performance, we incorporated RF and SVM methods in a cross-validation scheme, which is explained in Supplementary Material. We applied this cross-validation scheme for each parameter C and selected the combination of parameter C and number of features providing the max area under the curve (AUC). Using such number of features, we performed a receiver operating characteristic analysis.

#### 2.4.1 Predictions in the simulated disease progression

As we were also interested in estimating when alterations associated with dementia begin to be significantly apparent in the simulated progression process, a final SVM based on the identified features was fitted using all ADNI samples, and the resulting predictive model was independently tested on each NKI simulated stage (Fig. 1D). We then computed performance measures of AUC, sensitivity, specificity, and accuracy produced throughout the modeled progression span and evaluated the significance of these values. We used one-tailed binomial tests to evaluate the significance of sensitivity, specificity, and accuracy [33] and the nonparametric Wilcoxon rank-sum test to evaluate the significance of AUC values [34]. We declared results as significant at P<.05. We further used false discovery rate–adjustment procedures [35] with q=0.05 to control for multiple comparisons across stages within each performance measure. In all cases, we reported values of sensitivity, specificity, and accuracy at the threshold for classification, providing the best tradeoff between sensitivity and specificity.

## 3 Results

### 3.1 Diagnosis of Alzheimer's dementia

**Fig. 2:**
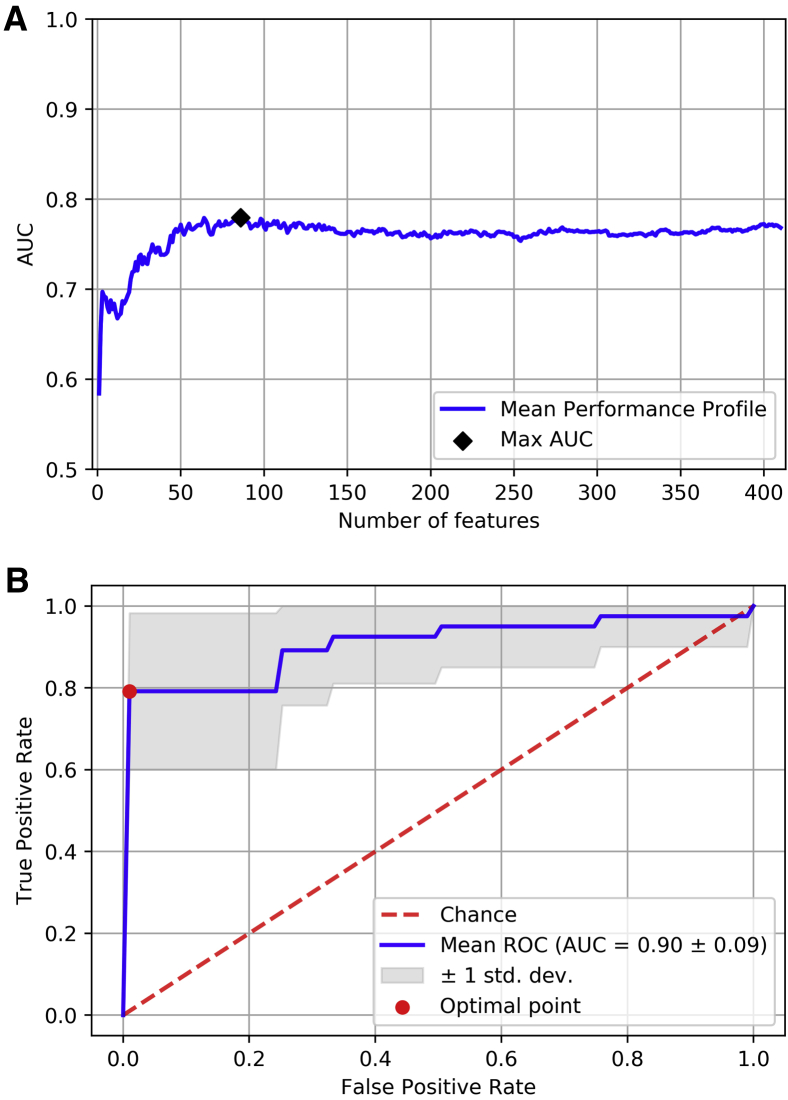
Machine learning analysis to predict dementia based on centrality metrics using the ADNI data set. (A) The mean performance profile computed as a function of the number of features included in the training process of radial SVMs (with the parameter C=100 controlling the complexity of the decision boundary). The most relevant features are identified at smaller values of the x-axis, so features were added progressively according to their relative importance. The max AUC, black diamond, was achieved with the first 86 most important features. Supplementary Fig. 1 shows the histogram of the AUC index at this point. (B) The mean ROC curve generated from the features identified in (A). Sensitivity, specificity, and accuracy indices were computed at the optimal point (red circle). Such a point was identified as the closest point (in terms of Euclidean distance) on the ROC curve to the point defined by a true positive rate of 1 and a false positive rate of 0. Abbreviations: ADNI, Alzheimer's Disease Neuroimaging Initiative; AUC, area under the curve; ROC, receiver operating characteristic; SVM, support vector machine.

We first analyzed the ADNI data set with machine learning tools to explore whether structural networks inferred from diffusion MRI predict Alzheimer's dementia. Using the cross-validation approach, we achieved the best mean classification performance when using radial SVMs with C=100 and the first 86 most relevant features according to the ranking generated with RF algorithm (max *AUC* = 0.78 ± 0.16, mean ± standard deviation) (Fig. 2A), so the remaining 324 network features did not provide further information for diagnosis. We then analyzed the mean receiver operating characteristic curve generated with the selected features. With the 86 more relevant features selected, we further evaluated their classification value with a 10-fold cross-validated SVM (with optimal parameters). At the optimal threshold for classification, the mean sensitivity was 79.16% (*P* = .147 × 10^−3^), mean specificity 98.9% (*P* = 1.81 × 10^−12^), and mean accuracy 89.07% (*P* = 6.9 × 10^−13^), substantially higher than the empirical distribution (50%) (Fig. 2B).

**Fig. 3:**
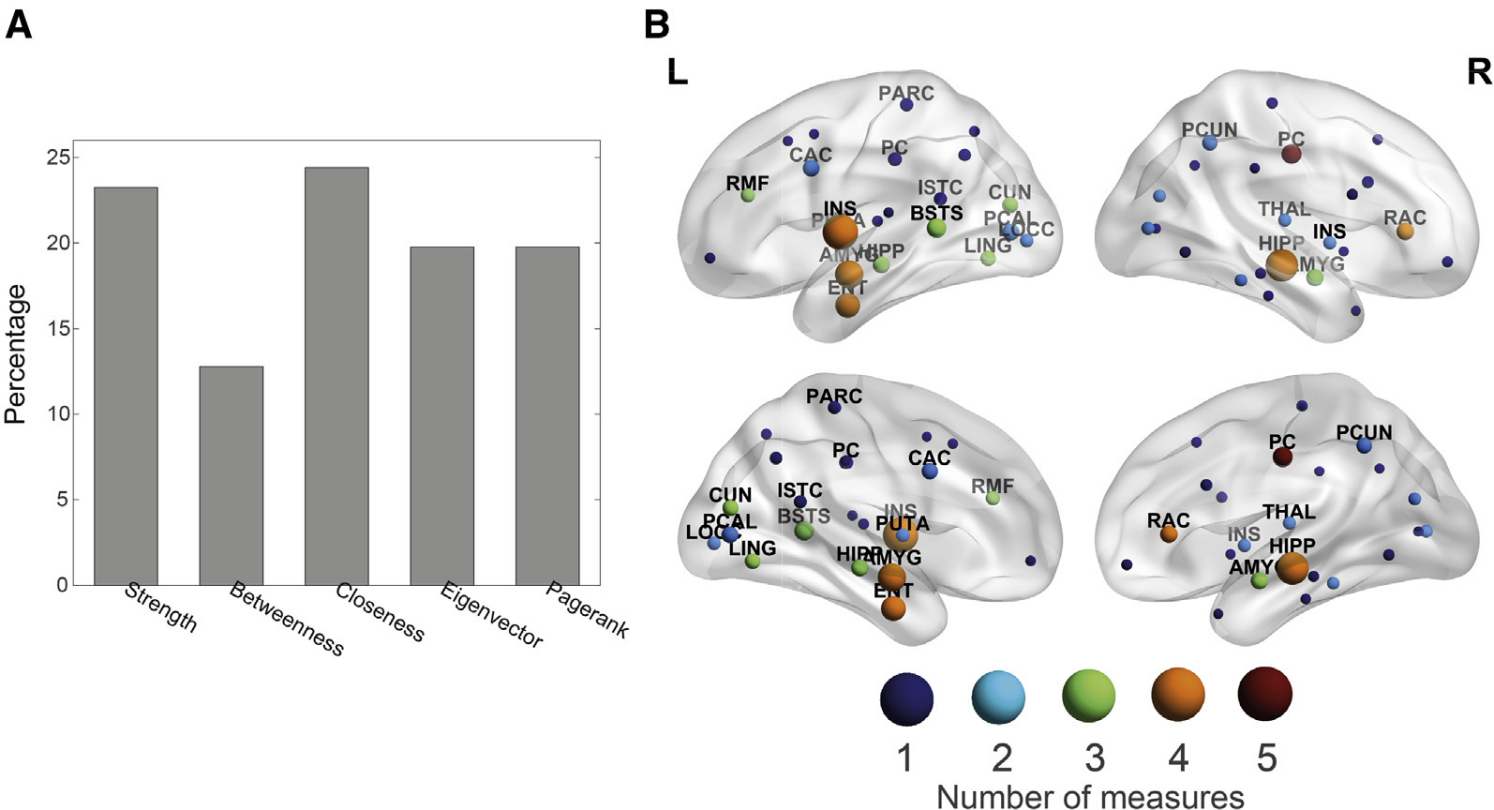
Identification of relevant centrality measures and brain regions. (A) A bar chart representing the proportion of each centrality measure that was included in the final set of selected features. (B) Brain map representing the most predictive regions to classify between dementia and healthy control. Lateral and medial views are shown on the top and bottom rows, respectively. The node size is proportional to its relative importance, which was computed from the feature ranking. Node color encodes the number of centrality measures included in the final set of features referring to that specific region. Abbreviations are shown for those regions having a relative importance above the median, and their definitions are given in Supplementary Table 1.

Then, we identified the most discriminative centrality measures and brain regions providing such performance. Strength and closeness centrality together account for almost half of the relevant features (23.25% and 24.42%, respectively). Measures of eigenvector and pagerank centrality have a lesser influence for classification but still represent a considerable proportion of the retained features (19.77% in both cases). Betweenness centrality is the least representative measure in the final set of selected features, accounting for the remaining 12.79% (Fig. 3A; see also Supplementary Table 2 for a detailed listing of the selected features). To identify the most characteristic areas of dementia, we summed the feature importance scores of the network features referring to the same area, obtaining the relative importance of individual areas (Fig. 3B; see also Supplementary Material).

The applied cross-validation scheme allows computing the classification performance while controlling for overfitting but does not provide a single predictive model. We, therefore, fitted a final SVM based on the 86 selected features using all ADNI samples, and this model was tested on the NKI simulated data set.

### 3.2 Predictions in the simulated disease progression

**Fig. 4:**
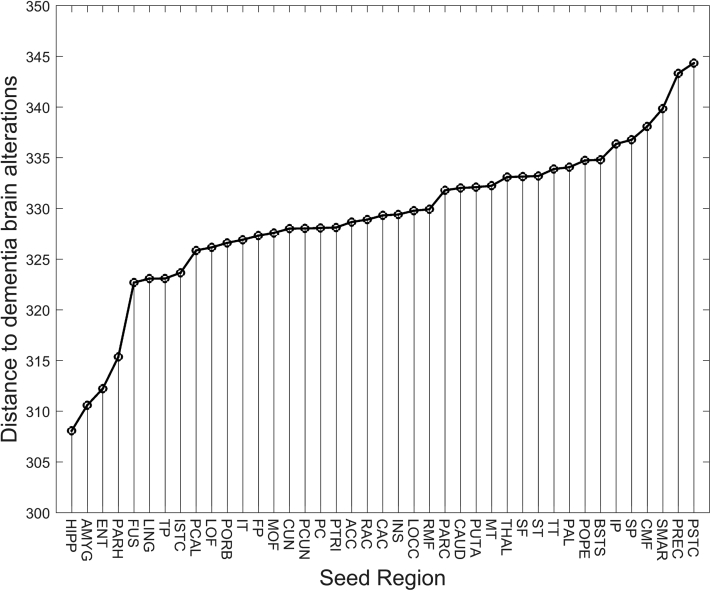
Parameter optimization of the disease progression model. Upon applying dynamical simulations using the NKI data set, this figure assesses which seed region is most likely to reproduce the differences in node strength measured between ADNI control and ADNI patients. For each region, we explored the parameter space given by α and $\beta^{AD}$ to find the minimum cost function (see subsection 2.3.3). The hippocampus (HIPP) turned out to be the very first region affected by Alzheimer's disease. Abbreviations: ADNI, Alzheimer's Disease Neuroimaging Initiative; NKI, Nathan Kline Institute-Rockland Sample. Expansions for the seed region abbreviations are given in Supplementary Table 1.

We incorporated the NKI data set to simulate 16 disease stages and their corresponding age-matched normal phases, relative to individuals following an AD-related pathological (NKI-II group) and healthy (NKI-I group) pathway. For the pathological pathway, different trajectories were simulated to identify the model parameters resembling dementia brain patterns at simulated stage 0 compared with the ADNI *A−* control and *A+* Alzheimer's patient data set. Upon parameter optimization, the hippocampus was identified as the most likely site of origin of AD (with α=0.4 and $\beta^{AD}=0.025$). Other regions, especially the amygdala and entorhinal cortex, are plausible seed candidates where AD first originates (Fig. 4).

**Fig. 5:**
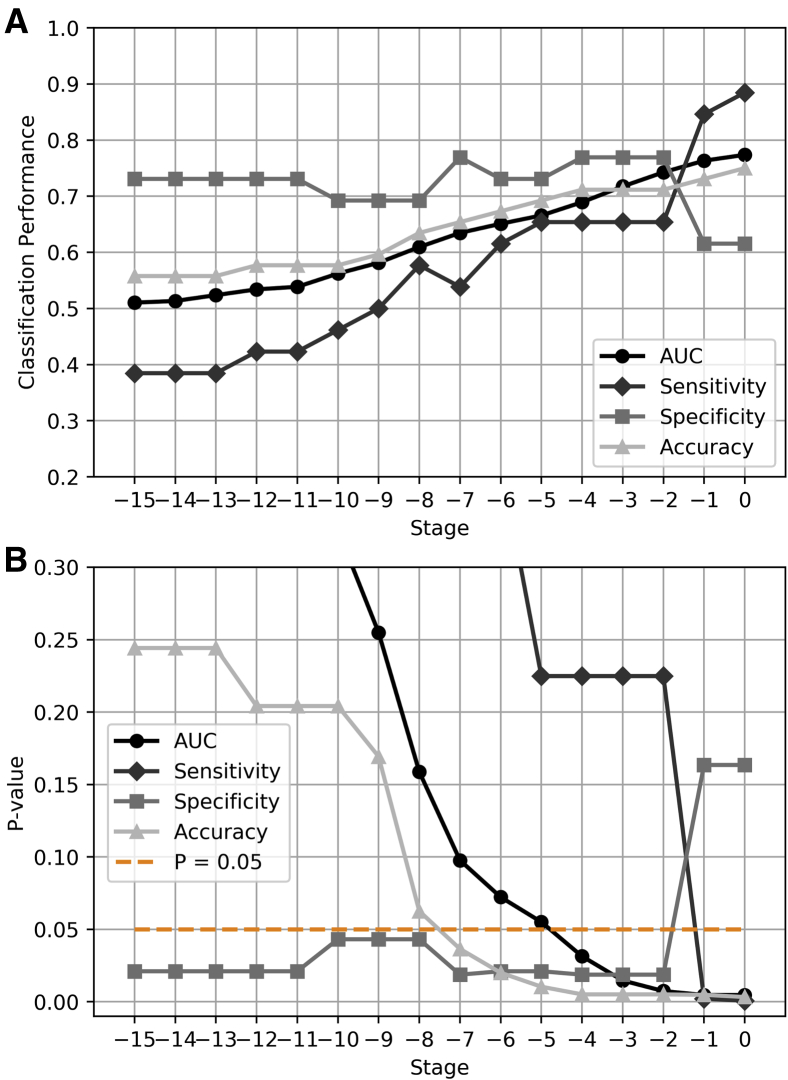
Classification across simulated stages using the hippocampus as the seed region. (A) A final SVM based on the most discriminative features of dementia (Fig. 3A) was fitted using all ADNI subjects. This predictive model was then used to classify between simulated patients and simulated controls across the progression span produced with the NKI data set. Performance measures of AUC, sensitivity, specificity, and accuracy were computed at each stage. (B) Based on the AUC and accuracy indices, network alterations associated with dementia begin to manifest structurally at the simulated stage 7. Abbreviations: ADNI, Alzheimer's Disease Neuroimaging Initiative; NKI, Nathan Kline Institute-Rockland Sample; AUC, area under the curve; SVM, support vector machine.

Using the hippocampus as the seed region, we produced connectivity matrices either representing individuals normally aging or individuals developing AD, and the ADNI classifier, which was only based on the relevant features associated with dementia (Fig. 3A), was evaluated on these structural networks (Fig. 5A). To estimate the cutoff stage from which network alterations associated with dementia begin to manifest structurally, we calculated the significance of the classification performance measures throughout the simulated progression span. Interestingly, we observed that the selected centrality metrics still capture significant dementia alterations at the simulated stage 7 (Fig. 5B). The classification performance progressively improved as AD progressed (Fig. 5A), achieving more accurate predictions as the effect of the disease becomes more prominent. Note that this improvement comes from the rise in the sensitivity index, whereas specificity remains relatively stable across simulated stages until stages 1 and 0, at which specificity decreases. At very early stages, both simulated controls (NKI-I subjects) and simulated patients (NKI-II subjects) are generally classified as controls, when the impact of dementia on brain connectivity is yet subtle.

We finally explored the evolution of predictions when the remaining 40 brain areas served as the seed region to initiate the propagation of the disease factor (Supplementary Fig. 3). Those regions are most likely the site of origin of the disease (Fig. 4) and offer a gradual increase in classification performance throughout simulated stages. When AD simulation originated from the amygdala and the entorhinal cortex, performance measures were similar to the optimal seed (the hippocampus).

## 4 Discussion

In this study, we propose an approach grounded on network neuroscience to assess the potential of structural brain networks as measured with diffusion MRI in prodromal dementia. The main findings uncovered in this work suggest that centrality measures of diffusion MRI networks are informative for Alzheimer's dementia diagnosis; the most discriminative network features are largely associated with medial temporal and subcortical brain regions, as well as posterior structures of the default mode network (DMN) and occipital areas; the hippocampus is the likely origin of AD; and pathophysiological alterations associated with dementia become significantly apparent at the simulated stage 7, presumably before meeting diagnostic criteria for clinical dementia.

### 4.1 Prediction of AD using real-world data

It has been suggested that the pathophysiological processes in AD largely disturb hub regions [15], [16]. Here, we have used sophisticated multivariate techniques to predict dementia based on 86 features quantifying the centrality of individual nodes, obtaining significant classification performance: (1) 79.16% mean sensitivity; (2) 98.9% mean specificity; and (3) 89.07% mean accuracy.

Earlier investigations have developed classifiers to distinguish patients with AD in different phases or stages from healthy controls, as reviewed in Supplementary Material. A comparison between classifiers across studies is not straightforward due to variations in predictors, data sets, or patient's disease stage. Importantly, to the best of our knowledge, our work is the first to mechanistically model brain network degeneration for early Alzheimer's dementia diagnosis.

### 4.2 Discriminative brain signatures of dementia

There is literature supporting a stereotypical pattern of neurodegeneration in AD which is associated with tau pathology of Braak staging [36], [37]. At early stages, atrophy largely occurs in the entorhinal cortex, hippocampus, and posterior structures of the DMN [36], [38], [39]. From these regions, atrophy then extends to the lateral temporal cortex, dorsal parietal, and frontal cortex. Finally, sensorimotor and visual cortices are affected at late stages [40], [41]. In this work, the relevant features extracted using the ADNI data set uncovered a set of brain regions, such as the entorhinal cortex, hippocampus, and other subcortical structures, as well as posterior structures of the DMN and the occipital lobe, that are highly predictive of dementia. Our results are in line with previous literature, with the entorhinal and subcortical structures having a greater weight for classification.

### 4.3 Spreading processes and AD

We modeled the degradation of structural connectivity based on the disconnection hypothesis [13], [14]. The most plausible scenario was when the disease is initiated in the hippocampus, as well as in the entorhinal cortex and amygdala. Numerous studies have shown levels of atrophy and functional disruption in the hippocampus and the entorhinal cortex at very early stages of the disease [13], [38], [42]. The initial Braak stages are also characterized by the presence of tau proteins in these areas, which in turn extend to the amygdala [37]. Interestingly, atrophy and amyloid-β (Aβ) patterns in AD can be explained using brain models and disease agents that propagate in a prion-like manner (see [9] and Supplementary Material).

### 4.4 Network alterations in the simulated disease progression

We found that a classifier based on centrality features using the ADNI data set provides significant predictions in the simulated progression process. In particular, AUC and accuracy indices become significant at simulated stages 4 and 7, respectively. At this stage, clinical symptoms may not yet be detectable: assuming that AD progresses continuously at a constant rate, our model suggests that alterations associated with dementia could be detected up to 7 years before the diagnosis established in the ADNI database. Hence, our model simulations suggest a valuable opportunity for risk assessment and early diagnosis. Moreover, our simulations highlight the possibility that a neural structure may be significantly compromised before cognitive deficits become evident enough for a clinical diagnosis, possibly due to compensatory plasticity [40]. Nevertheless, AD progresses at different rates in actual subjects and a direct link between simulated stages and years of progression might be subject to large intersubject variation.

### 4.5 Limitations

The final set of discriminative brain signatures included medial temporal and subcortical structures, which are thought to be disrupted at early stages [43]. However, it is important to acknowledge that the relevant features were extracted from ADNI patients with dementia, which is not a prodromal phase. Thus, future research could incorporate data from patients at earlier stages (e.g., before or during the mild cognitive impairment phase and prodromal dementia) for a definitive validation. For instance, our model could be tested retrospectively for patients who were scanned within the UK Biobank project [44] before the disease onset. Importantly, one should include individuals with an age range in agreement with the simulated period because AD is an age-dependent disorder.

On the technical side, NKI and ADNI structural connectivity was reconstructed using deterministic tracking. Although probabilistic tracking could lead to a more accurate reconstruction, we used deterministic tracking in line with earlier studies in the NKI data set [20]. Also, owing to the FMRIB Software Library version that was used at the time of analysis, the old eddy_correct tool rather than the new more accurate eddy tool was used for eddy current and movement correction that could influence the reconstruction.

As patients might be affected by cerebrovascular diseases [45] and other copathologies such as alpha-synuclein [46], the diagnosis could be enhanced by accounting for these comorbidities. Furthermore, as new information about the selective neural vulnerability in AD is gathered [47], and more complex models could be implemented to mimic mechanisms of degeneracy and reserve [11]. This would also enable a more mechanistic modeling of aging, as the aggregation of misfolded proteins in the brain is part of the normal aging process [48].

### 4.6 Conclusion

Computational models of disease progression based on the connectome are an approach to discover risk factors and biomarkers of brain network diseases [49]. We have used such a model to study the progression from networks of healthy controls to networks showing features of patients with AD. This highlighted centrality as an early risk factor of developing dementia. Overall, our work identifies potential anatomical origins of AD and suggests a diffusion MRI-based biomarker for early diagnosis.

**Research in Context**

1. Systematic review: We reviewed the literature using PubMed and Google search engine. Nodes with high centrality are disrupted in Alzheimer's disease (AD). Computational models of spreading processes based on the structural connectome have been capable of explaining the macroscopic atrophy and Aβ aggregation in AD.
2. Interpretation: We have proposed a mechanistic computational model that simulates progressive structural connectivity degradation caused by AD to assess the extent to which network alterations associated with dementia begin to manifest structurally. Our model simulations suggest that centrality is not only disrupted in AD but also at early stages.
3. Future directions: This article proposes centrality as a potential biomarker before the onset of symptoms and further research to assess the ability of the model to explain connectivity degradation in real-world patients with prodromal dementia; the influence of copathologies for AD prediction; and the importance of accounting for mechanism of degeneracy and reserve.
