# Supplementary material for: Structural connectivity centrality changes mark the path toward Alzheimer's disease
Source: Alzheimers Dement (Amst). 2019 Jan 18;11:98–107. doi: 10.1016/j.dadm.2018.12.004 (PMC6350419; doi:10.1016/j.dadm.2018.12.004)
Supplement: Supplementary Material [file mmc1.docx]

**Supplementary Material**

**Introduction**

Network neuroscience allows the formal investigation of neural phenomena by modeling the brain as a complex network of areas or regions (network nodes) that are connected (network links/edges) [1]. There is considerable literature describing the topology of AD networks using different acquisition techniques [2-4]. Previous investigations have approached AD progression by leveraging computer models simulating the spreading dynamics of a disease factor or agent across structural connections measured with diffusion MRI [5, 6]. Furthermore, machine learning algorithms have gained prominence in neuroimaging because of their ability to decode brain signatures and predict clinical outcomes [7-9]. In AD, different types of features can be extracted: atrophy-related measures [10-12]; features extracted from amyloid imaging [13]; predictors based on functional [14] and structural brain networks [15-17]; as well as multimodal descriptors describing both imaging and clinical data [18, 19].

**Methods**

**Participants and MRI acquisition protocol**

The ADNI was launched in 2003 as a public-private partnership, led by Principal Investigator Michael W. Weiner, MD. The primary goal of ADNI has been to test whether serial MRI, PET, other biological markers, and clinical and neuropsychological assessment can be combined to measure the progression of MCI and early AD. The primary goal of the NKI is to generate a large scale, extensively phenotyped dataset for discovery science. For the selected 39 normal controls and 39 age- and sex-matched AD patients from the ADNI cohort, the Florbetapir standard uptake value ratio (SUVR) was retrieved from the ADNI database website (<http://adni.loni.usc.edu/>). Recommended threshold values for confirmation of AD is an SUVR >= 1.1 [20], and this threshold was used to select the ADNI amyloid-β positive (*A+*) group. For healthy controls, however, a less strict threshold of SUVR <= 1.2 was chosen to select the ADNI amyloid-β negative (*A-*) control group. Participants from the NKI database were selected as healthy controls, and executive functioning results from this group are provided in Supplementary Table 3.

All subjects underwent T1-weighted structural MRI and diffusion MRI scanning. For the ADNI dataset, MRI data were acquired on 3 Tesla GE Medical Systems scanners (Boston, Massachusetts, USA) at different acquisition sites across North America. Diffusion images were recorded applying the following parameters: matrix size = 256 × 256; voxel size = 2.7 × 2.7 × 2.7 mm^3^; 59 slices; 5 images with no diffusion sensitization and 41 diffusion directions with *b*-factor of 1000 s mm^-2^. More imaging details can be found at <http://adni.loni.usc.edu/wp-content/uploads/2010/05/ADNI2_GE_3T_22.0_T2.pdf>. For the NKI dataset, MRI data were measured with a 3 Tesla Siemens MAGNETOM TrioTim syngo scanner (Erlangen, Germany). In this case, diffusion images were recorded applying the following parameters: matrix size = 128 × 128; voxel size = 2 × 2 × 2 mm^3^; 58 slices; 12 images with no diffusion sensitization and 64 diffusion directions with *b*-factor of 1000 s mm^-2^. More imaging details can be found at <http://fcon_1000.projects.nitrc.org/indi/pro/nki.html>.

**Preprocessing of MRI data and estimation of structural networks**

Structural images were preprocessed using *FreeSurfer v5.3* (<http://surfer.nmr.mgh.harvard.edu/>) as described by Wang *et al.*[21]. The preprocessing stream of *FreeSurfer* fundamentally performs skull stripping, intensity normalization, subcortical region segmentation, grey and white matter segmentation, and cortical surface extraction for subsequent labeling. The resulting subdivision of the cerebral cortex based on the Desikan/Killiany atlas [22] was combined with subcortical structures to generate a brain parcellation containing 34 cortical and 7 subcortical regions for each hemisphere (see Supplementary Table 1 for a detailed listing of the included regions). The same parcellation has been previously used in the context of AD [10, 23].

Raw diffusion data were downloaded from ADNI in DICOM data format. Images were then converted to NIfTI data format using the *dcm2niix* tool available in *MRIcroGL* (<https://www.nitrc.org/plugins/mwiki/index.php/dcm2nii:MainPage>) and subsequently preprocessed with *FSL v5.0* (FMRIB Software Library, <https://fsl.fmrib.ox.ac.uk/fsl/fslwiki/>)[24]. *Dcm2niix* automatically provides a table that stores the diffusion gradient vector for each acquired volume. Using the first b_0_ image as a reference, diffusion images were corrected for eddy current and head motion distortions by using the *eddy_correct* tool [25]. Gradient vectors were rotated accordingly using the *fdt_rotate_bvecs* function. Next, brain extraction was performed with *BET* [26] before reconstructing diffusion tensors at each voxel through the *Diffusion Toolkit* suite (<http://trackvis.org/dtk/>). Deterministic fiber tracking was carried out by applying the *FACT* method [27]. Single seed points to reconstruct streamlines were placed in the center of each voxel belonging to the brain and tracking was terminated for curvatures greater than 35º.

For network generation, streamlines were first aligned with the brain parcellation. The first skull-stripped b_0_ image was linearly registered to the preprocessed structural image using *FSL flirt* with six degrees of freedom [28]. Next, the resulting transformation matrix was applied to every streamline using the *track_transform* function included in *Diffusion Toolkit* software. The *UCLA Multimodal Connectivity Package* (<https://github.com/jbrown81/umcp>) was used to obtain an 82 × 82 weighted connectivity matrix for each subject. Structural interactions were quantified by counting the number of streamlines connecting two regions (self-connections were not considered). Finally, only streamlines with either endpoints terminating at the two regions and greater than 5 mm were included.

**Normal aging model**

The *subset selection* approach involves fitting a separate least squares regression for each possible combination of the *p* predictors and selecting the model that is the “best” regarding accuracy and complexity. As $p=4$, a total of 16 different models were fitted containing all possible combinations. Next, among those models containing the same number of variables, the one giving the greater coefficient of determination, $R^{2}$, was chosen. This step retained five different models, which contained zero (*i.e.*, a model predicting merely the sample mean through $\beta_{0}^{ij}$), one, two, three and four predictors. Finally, the models were compared by using the Akaike information criterion (AIC), and the model providing the smallest AIC was selected to predict $w_{ij}$. Note that we did not compute any *P*-value in this analysis, since our objective was to maximize the ability of the model to predict the response rather than explain the association between variables and response.

**Dynamical simulations**

To simulate disease trajectories, we pre-specified different alpha values ranging from 0 to 1.5 in increments of 0.05 and beta values ranging from 0 to 0.15 in increments of 0.005. These ranges were chosen upon empirically testing possible parameter values to reproduce the differences in structural connectivity between ADNI controls and ADNI patients. For each combination of alpha and beta values, a different brain region in both hemispheres (41 seed regions) was deemed as origin to initiate the progression. By using matrices as input data and for each parameter combination within the set $\left\{ seed, \alpha,\beta^{AD} \right\}$, we numerically computed model solutions of equations (2), (3) and (5) in *MATLAB 2016a* (MathWorks Inc.), with a step size of 0.1. This process produced a set of simulated structural networks based on the changes in connection strength either caused by the process of normal aging or by AD.

**Feature extraction and machine learning analysis**

For model evaluation, the ADNI dataset was split into training and test subsets by using the 10-fold cross-validation technique, ensuring that classes were balanced within each subset. In each iteration, an RF was fitted by using the training subsets, giving rise to a ranking encoding the relative importance of each feature. To identify the minimum number of features, providing good predictions, radial SVMs were then trained by adding features progressively. That is, a first radial SVM was computed using the most relevant feature (according to the foregoing ranking). Next, a second radial SVM was obtained using the two most important features, sequentially adding features until all features were considered. Each of the 410 radial SVMs was evaluated on the test subset and the area under the curve (AUC) of the receiver operating characteristic (ROC) was recorded as a function of the number of features, producing a performance profile. The whole process was further repeated 10 times. Performance profiles and feature importance scores generated across the 100 iterations were averaged.

**Pathological assessment of the NKI and ADNI databases with VBM**

In order to assess the presence of AD-related pathology within the NKI data set, we performed voxel-based morphometry (VBM) analyses with SPM12 [29]. First, we studied differences between ADNI *A+* Alzheimer’s patients and the joint group of NKI and ADNI *A-* healthy controls with a two-sample t-test design, and with age, sex, and total intracranial volume (TIV) as covariates. Additionally, a dichotomous covariate to account for study differences (ADNI, NKI) was also included. Afterward, we wanted to test if NKI participants were structurally different to the ADNI *A-* healthy controls. Because these two groups belonged to different studies, a direct comparison was not possible. However, we converted the previous SPM design into a one-sample t-test comprising the ADNI *A+* Alzheimer’s patients, ADNI *A-* controls and the NKI participants, and regressed all four covariates (age, sex, TIV, and study) in order to obtain residual images. This procedure allows us to normalize all images while accounting for study differences and the other covariates. We then performed a standard two-sample t-test between the ADNI *A-* control and NKI participant residual images (without covariates).

**Results**

**Diagnosis of Alzheimer’s dementia**

The entorhinal cortex and subcortical structures, such as the hippocampus, insula, amygdala, putamen, and thalamus, are highly discriminative in detecting Alzheimer’s dementia, especially in the left hemisphere. Other regions playing an important role to distinguish between controls and patients are the posterior cingulate and precuneus cortices, with the right hemisphere showing a greater relevance. The left occipital lobe and surrounding areas (the isthmus division of the cingulate cortex and the banks of the superior temporal sulcus) are also critical for diagnostic separation. The rostral anterior division of the right cingulate cortex, along with the caudal anterior division of the left cingulate cortex, the left paracentral lobule, and the rostral division of the left middle frontal gyrus capture relevant effects. Other regions from frontal and parietal lobes are further relevant for classification, although to a lesser extent.

**VBM analysis of the NKI and ADNI data sets**

The VBM analyses revealed that the ADNI *A+* patient group presented with lower gray matter volume at regions highly associated with AD pathology, e.g., the middle temporal gyri. Gray matter volume at these regions was significantly lower than healthy controls (ADNI *A-* controls and NKI participants, p-value < 0.05, FWE corrected for multiple comparisons), supplementary Fig. 4A. The comparison for AD > Controls did not show significant differences.

When comparing the residual images between ADNI *A-* controls and NKI participants, the ADNI *A-* group showed greater gray matter volume than the NKI group within a small cluster at the right hippocampus (ADNI > NKI, p < 0.05, FWE corrected for multiple comparisons), supplementary Fig 4B. The reverse contrast (ADNI< NKI) did not show significant differences between groups.

**Discussion**

**Prediction of AD using real-world data**

Klöppel *et al.* [1*2*] developed a classifier based on atrophy-related measures, and they reported 89% accuracy in separating patients with mild dementia from matched controls. Based on characteristics extracted from the gray matter, Magnin *et al.* [11] achieved values of 94.5%, 91.5% and 96.6% for accuracy, sensitivity and specificity, respectively, in detecting dementia. However, their dataset comprises a rather small number of patients (5 male / 11 female), and so it is problematic to generalize these high accuracies. Using connection weights as features extracted from diffusion MRI, Zhan *et al.* [17] systematically compared different tractography algorithms for classification. They concluded that the ability to classify between dementia and control conditions was higher than comparisons between dementia and MCI conditions. By contrast, Ebadi *et al.* [15] developed an ensemble classification module based on graph metrics to perform classification across diagnostic groups. These authors obtained better classification performance when classifying between dementia and MCI conditions (accuracy of 83.3%) than when distinguishing patients with dementia from healthy controls (accuracy of 80%). Based on connection weights, Shao *et al.* [16] achieved classification accuracies greater than 95% using data from 17 patients. Other studies have developed predictive models to estimate which MCI individuals will develop dementia within a certain time window. For example, a recent study using amyloid imaging [13] has reported values of 84% and 0.91 for accuracy and AUC, respectively, when assessing the progression to dementia within two years.

**Spreading processes and AD**

There is a correspondence between the AD-associated features identified in this work and relevant patterns identified by other researchers. Along these lines, eigenmodes derived from a network diffusion model recapitulate atrophy patterns measured in AD and behavioral frontotemporal dementia [5]. This model relies on a diffusive mechanism where a disease agent accumulates in brain areas giving rise to atrophy. The same authors extended their approach by predicting both atrophy and metabolism in AD. Iturria-Medina *et al.* [6] describe a spreading model to reproduce regional amyloid-β patterns measured with PET imaging. Their model also incorporates a term capturing mechanisms of amyloid-β clearance. These findings are further supported by recent work showing that intrinsic functional connectivity cannot explain the tendency for strongly connected nodes to have more tau pathology [30].

**NKI and ADNI group gray matter integrity**

The main purpose of the VBM analysis was to study the presence of AD pathology within the NKI sample which we used to mathematically model healthy aging and AD progression. Here we used gray matter volume as a proxy of AD pathology. It is well documented that AD targets the middle temporal gyri, and specifically the hippocampal tissue is severely affected by this disease. We confirmed this for the selected AD patients who were amyloid positive *A+;* These participants had lower gray matter volume than the control group at the frontal cortex, cingulate gyrus, and temporal cortices, with the lowest volume observed at both hippocampi. On the contrary, we did not observe such differences between the ADNI *A-* controls and NKI participants, which suggests that AD pathology in this latter group is minimal.

**Figures**

**
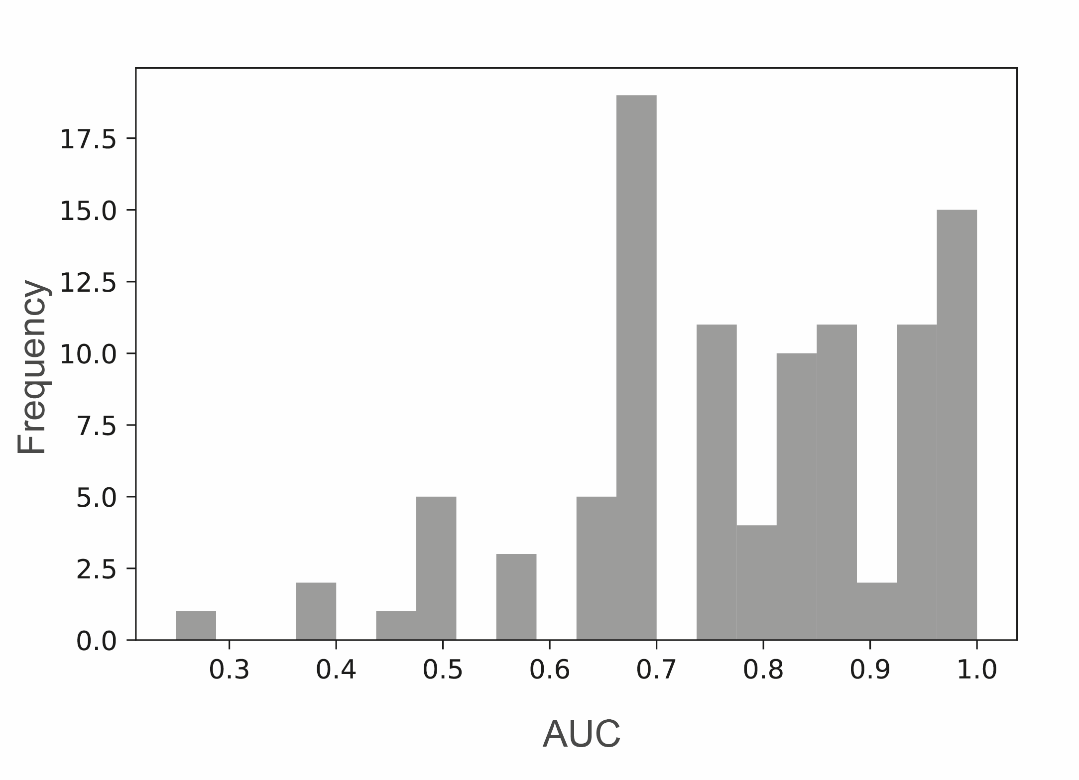
**

**Supplementary Fig. 1.** Histogram of the AUC index based on the first 86 most relevant features. This figure presents the 100 AUC values resulting from the cross-validation scheme when distinguishing ADNI patients from ADNI controls by using the number of features that yielded the greatest mean AUC index.

**
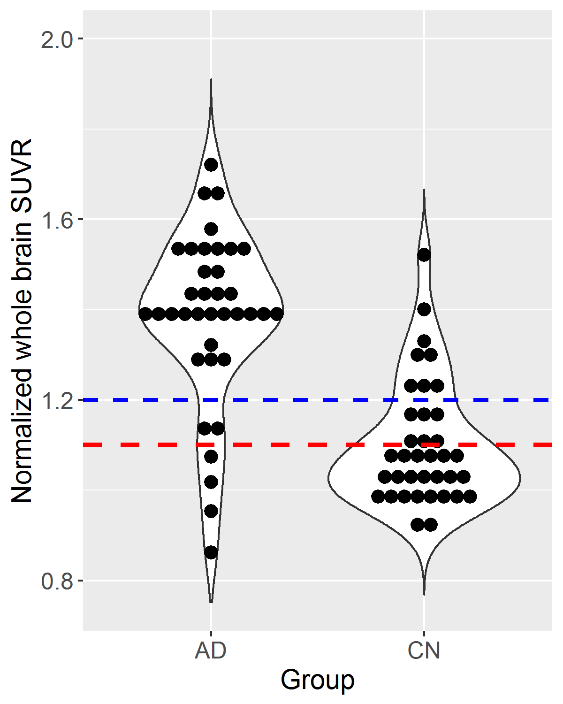
**

**Supplementary Fig. 2.** Florbetapir standard uptake value ratios (SUVR) for the ADNI cohort. The ADNI amyloid-β positive group (*A+* AD) was selected from those clinically diagnosed AD patients with an SUVR >= 1.1 (red line). The ADNI amyloid-β negative control group (*A-* CN) was selected from the ADNI participants diagnosed as healthy controls with an SUVR <= 1.2 (blue line). In other words, healthy controls with very high amyloid uptake as well as AD patients with low amyloid uptake were excluded from modeling and simulation steps. See also Table 1 in the main manuscript.

**
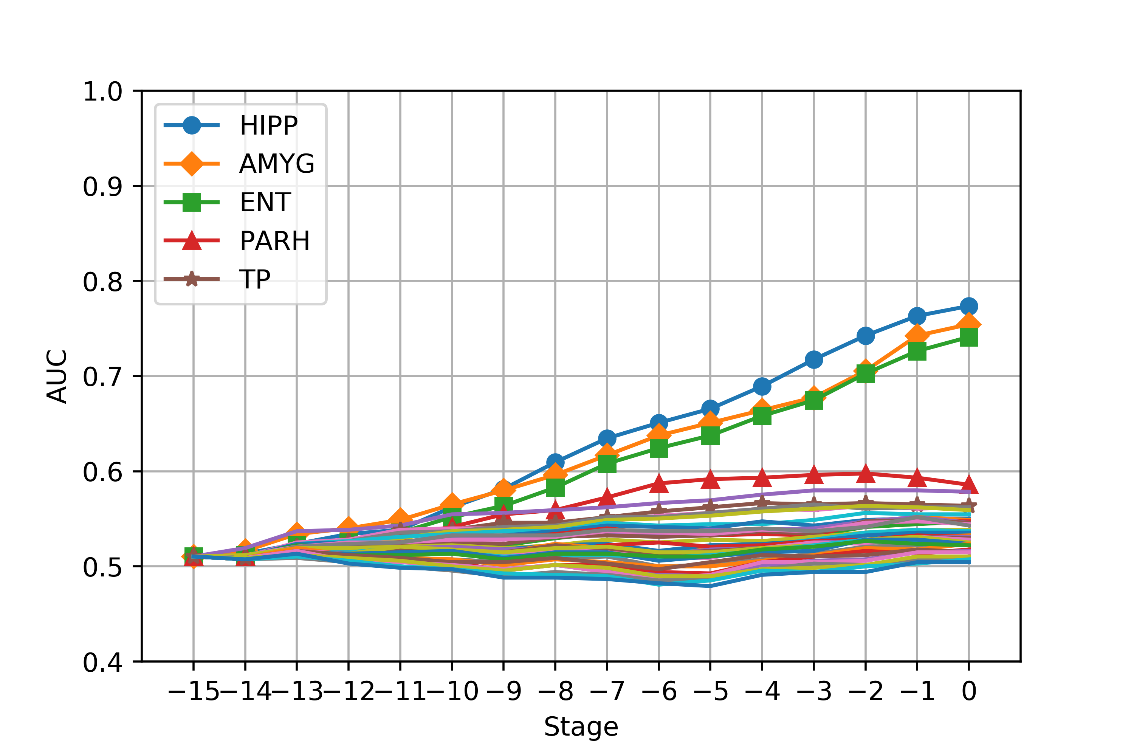
**

**Supplementary Fig. 3.** Classification performance along the disease progression when using different seeds. The AUC index was calculated at each simulated stage when individual regions were set as the origin to initiate the propagation of the disease factor. Hippocampus, amygdala and entorhinal cortex structures offered a similar roughly linear trend.

**
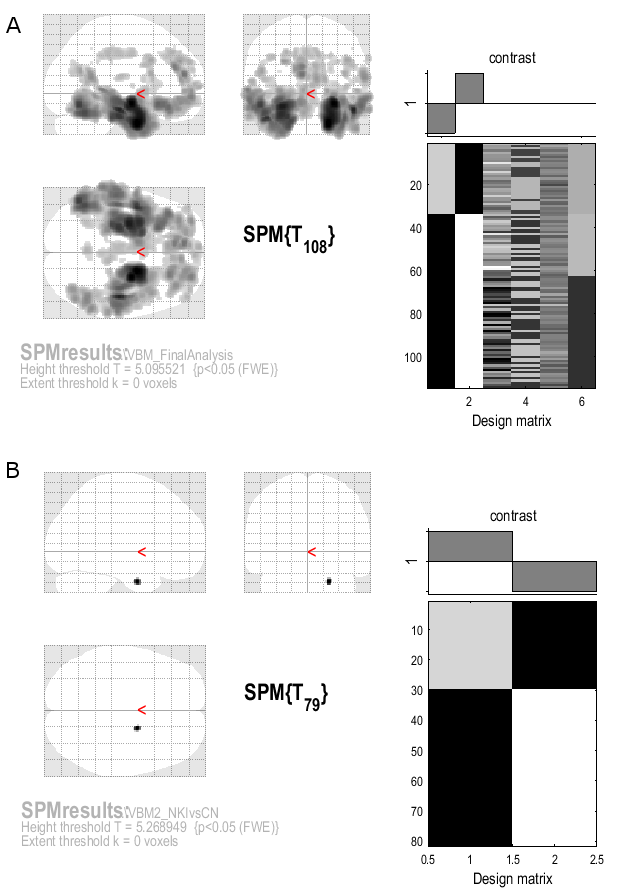
**

**Supplementary Fig. 4.** Voxel-based morphometry (VBM) analysis of the ADNI and NKI groups. A) VBM result for the ADNI AD amyloid-positive participants and the joint ADNI amyloid negative controls and NKI group (AD vs. Controls comparison). The contrast testing AD < Controls showed significant lower volume in AD patients compared to controls. B) Two sample t-test between ADNI amyloid negative controls and NKI participants after regressing age, sex, total intracranial volume, and study covariates. A small cluster at the right hippocampus was shown for the ADNI > NKI contrast.

**Tables**

**Supplementary Table 1.** Brain regions along with their respective abbreviations.

| **Brain structure** | **Abbreviation** | **Brain structure** | **Abbreviation** |
| --- | --- | --- | --- |
| **Banks of the superior temporal sulcus** | BSTS | **Posterior division of the cingulate cortex** | PC |
| **Caudal anterior division of the cingulate cortex** | CAC | **Precentral gyrus** | PREC |
| **Caudal division of the middle frontal gyrus** | CMF | **Precuneus cortex** | PCUN |
| **Cuneus cortex** | CUN | **Rostral anterior division of the cingulate cortex** | RAC |
| **Entorhinal cortex** | ENT | **Rostral division of the middle frontal gyrus** | RMF |
| **Fusiform gyrus** | FUS | **Superior frontal gyrus** | SF |
| **Inferior parietal cortex** | IP | **Superior parietal cortex** | SP |
| **Inferior temporal gyrus** | IT | **Superior temporal gyrus** | ST |
| **Isthmus division of the cingulate cortex** | ISTC | **Supramarginal gyrus** | SMAR |
| **Lateral occipital cortex** | LOCC | **Frontal pole** | FP |
| **Lateral division of the orbitofrontal cortex** | LOF | **Temporal pole** | TP |
| **Lingual gyrus** | LING | **Transverse temporal cortex** | TT |
| **Medial division of the orbitofrontal cortex** | MOF | **Insula** | INS |
| **Middle temporal gyrus** | MT | **Thalamus** | THAL |
| **Parahippocampal gyrus** | PARH | **Caudate** | CAUD |
| **Paracentral lobule** | PARC | **Putamen** | PUTA |
| **Pars opercularis of the inferior frontal gyrus** | POPE | **Pallidum** | PAL |
| **Pars orbitalis of the inferior frontal gyrus** | PORB | **Amygdala** | AMYG |
| **Pars triangularis of the inferior frontal gyrus** | PTRI | **Hippocampus** | HIPP |
| **Pericalcarine cortex** | PCAL | **Accumbens** | ACC |
| **Postcentral gyrus** | PSTC |  |  |

**Supplementary Table 2.** Most important features in predicting dementia. Each feature consists of a brain region (represented by its abbreviation, see Supplementary Table 1), the hemisphere which the brain region comes from, and the specific centrality measure associated with the region (*i.e.,* strength, betweenness, closeness, eigenvector or pagerank).

| **Position in the ranking** | **Region** | **Hemisphere** | **Centrality measure** |
| --- | --- | --- | --- |
| 1 | INS | left | pagerank |
| 2 | AMYG | left | closeness |
| 3 | HIPP | right | betweenness |
| 4 | INS | left | strength |
| 5 | ENT | left | closeness |
| 6 | INS | left | closeness |
| 7 | CAC | left | eigenvector |
| 8 | PC | left | eigenvector |
| 9 | HIPP | right | pagerank |
| 10 | HIPP | right | closeness |
| 11 | HIPP | right | strength |
| 12 | BSTS | left | strength |
| 13 | AMYG | left | strength |
| 14 | PARC | left | eigenvector |
| 15 | PCAL | left | eigenvector |
| 16 | ISTC | left | eigenvector |
| 17 | ENT | left | strength |
| 18 | PCUN | right | strength |
| 19 | PCUN | left | eigenvector |
| 20 | PC | right | eigenvector |
| 21 | AMYG | left | pagerank |
| 22 | BSTS | left | closeness |
| 23 | PCAL | left | closeness |
| 24 | CAC | right | eigenvector |
| 25 | LING | left | strength |
| 26 | PCUN | right | pagerank |
| 27 | ENT | left | pagerank |
| 28 | AMYG | right | strength |
| 29 | LOCC | left | closeness |
| 30 | HIPP | left | closeness |
| 31 | PARC | right | eigenvector |
| 32 | PUTA | left | pagerank |
| 33 | AMYG | right | pagerank |
| 34 | RAC | right | closeness |
| 35 | HIPP | left | strength |
| 36 | LING | right | betweenness |
| 37 | INS | left | betweenness |
| 38 | CUN | left | eigenvector |
| 39 | POPE | right | betweenness |
| 40 | HIPP | left | pagerank |
| 41 | PUTA | left | strength |
| 42 | LOCC | right | pagerank |
| 43 | FP | right | betweenness |
| 44 | AMYG | right | closeness |
| 45 | PARH | right | closeness |
| 46 | THAL | right | closeness |
| 47 | INS | right | strength |
| 48 | CUN | left | strength |
| 49 | THAL | left | closeness |
| 50 | FUS | right | strength |
| 51 | BSTS | left | pagerank |
| 52 | CUN | left | closeness |
| 53 | SP | left | eigenvector |
| 54 | FP | left | betweenness |
| 55 | RAC | right | strength |
| 56 | INS | right | pagerank |
| 57 | CUN | right | eigenvector |
| 58 | SF | right | closeness |
| 59 | PC | right | betweenness |
| 60 | CUN | right | strength |
| 61 | RAC | right | betweenness |
| 62 | THAL | right | eigenvector |
| 63 | LING | left | pagerank |
| 64 | RMF | left | pagerank |
| 65 | IT | right | closeness |
| 66 | ENT | left | betweenness |
| 67 | RMF | left | strength |
| 68 | SF | left | eigenvector |
| 69 | LOCC | left | strength |
| 70 | PCAL | right | closeness |
| 71 | RMF | left | closeness |
| 72 | CAC | left | betweenness |
| 73 | LOCC | right | strength |
| 74 | PC | right | strength |
| 75 | AMYG | left | eigenvector |
| 76 | LING | left | closeness |
| 77 | RAC | right | eigenvector |
| 78 | TT | left | pagerank |
| 79 | IP | right | betweenness |
| 80 | PC | right | closeness |
| 81 | SMAR | right | strength |
| 82 | ACC | right | pagerank |
| 83 | TP | right | closeness |
| 84 | PC | right | pagerank |
| 85 | FUS | right | pagerank |
| 86 | CMF | left | eigenvector |

**Supplementary Table 3**. Executive functioning data; NKI Rockland Sample participants.

| **D-KEFS Subtest** | **NKI-I (n=26)** | **NKI-II (n=26)** | ***P*-value** |
| --- | --- | --- | --- |
| **Sorting test** |  |  |  |
| Condition 1: Free sorting description score | 29.12 (9.8) | 32.8 (13.31) | 0.196 |
| Condition 2: Sort recognition description score | 31.52 (9.94) | 33.42 (13.2) | 0.484 |
| **Design fluency test** |  |  |  |
| Condition 1 filled dots: Total correct | 10.12 (3.29) | 10.46 (3.91) | 0.962 |
| Condition 2 empty dots only: Total correct | 9.68 (2.94) | 10.65 (3.0) | 0.327 |
| Design accuracy | 80.0 (10.67) | 80.0 (15.6) | 0.565 |
| Design fluency total correct | 32.64 (7.16) | 35.46 (7.11) | 0.444 |
| **Verbal fluency test** |  |  |  |
| Category fluency: Total | 42.0 (7.66) | 39.77 (10.64) | 0.274 |
| Category switching: total correct | 12.48 (2.98) | 13.23 (3.07) | 0.378 |
| Letter fluency: total correct | 40.4 (11.06) | 42.88 (11.2) | 0.396 |
| Category switching: switching accuracy | 10.16 (3.43) | 11.65 (3.83) | 0.133 |

**References**

[1] Bassett DS, Sporns O. Network neuroscience. Nature neuroscience. 2017;20:353.

[2] Lo C-Y, Wang P-N, Chou K-H, Wang J, He Y, Lin C-P. Diffusion tensor tractography reveals abnormal topological organization in structural cortical networks in Alzheimer's disease. Journal of Neuroscience. 2010;30:16876-85.

[3] John M, Ikuta T, Ferbinteanu J. Graph analysis of structural brain networks in Alzheimer’s disease: beyond small world properties. Brain Structure and Function. 2017;222:923-42.

[4] Xie T, He Y. Mapping the Alzheimer’s brain with connectomics. Frontiers in psychiatry. 2012;2:77.

[5] Raj A, Kuceyeski A, Weiner M. A network diffusion model of disease progression in dementia. Neuron. 2012;73:1204-15.

[6] Iturria-Medina Y, Sotero RC, Toussaint PJ, Evans AC, Initiative AsDN. Epidemic spreading model to characterize misfolded proteins propagation in aging and associated neurodegenerative disorders. PLoS computational biology. 2014;10:e1003956.

[7] Jie B, Wee C-Y, Shen D, Zhang D. Hyper-connectivity of functional networks for brain disease diagnosis. Medical image analysis. 2016;32:84-100.

[8] Taylor PN, Sinha N, Wang Y, Vos SB, de Tisi J, Miserocchi A, et al. The impact of epilepsy surgery on the structural connectome and its relation to outcome. NeuroImage: Clinical. 2018;18:202-14.

[9] Woo C-W, Chang LJ, Lindquist MA, Wager TD. Building better biomarkers: brain models in translational neuroimaging. Nature neuroscience. 2017;20:365.

[10] Desikan RS, Cabral HJ, Hess CP, Dillon WP, Glastonbury CM, Weiner MW, et al. Automated MRI measures identify individuals with mild cognitive impairment and Alzheimer's disease. Brain : a journal of neurology. 2009;132:2048-57.

[11] Magnin B, Mesrob L, Kinkingnéhun S, Pélégrini-Issac M, Colliot O, Sarazin M, et al. Support vector machine-based classification of Alzheimer’s disease from whole-brain anatomical MRI. Neuroradiology. 2009;51:73-83.

[12] Klöppel S, Stonnington CM, Chu C, Draganski B, Scahill RI, Rohrer JD, et al. Automatic classification of MR scans in Alzheimer's disease. Brain : a journal of neurology. 2008;131:681-9.

[13] Mathotaarachchi S, Pascoal TA, Shin M, Benedet AL, Kang MS, Beaudry T, et al. Identifying incipient dementia individuals using machine learning and amyloid imaging. Neurobiology of aging. 2017;59:80-90.

[14] Chen G, Ward BD, Xie C, Li W, Wu Z, Jones JL, et al. Classification of Alzheimer disease, mild cognitive impairment, and normal cognitive status with large-scale network analysis based on resting-state functional MR imaging. Radiology. 2011;259:213-21.

[15] Ebadi A, Dalboni da Rocha JL, Nagaraju DB, Tovar-Moll F, Bramati I, Coutinho G, et al. Ensemble classification of Alzheimer's disease and mild cognitive impairment based on complex graph measures from diffusion tensor images. Frontiers in neuroscience. 2017;11:56.

[16] Shao J, Myers N, Yang Q, Feng J, Plant C, Böhm C, et al. Prediction of Alzheimer's disease using individual structural connectivity networks. Neurobiology of aging. 2012;33:2756-65.

[17] Zhan L, Zhou J, Wang Y, Jin Y, Jahanshad N, Prasad G, et al. Comparison of nine tractography algorithms for detecting abnormal structural brain networks in Alzheimer’s disease. Frontiers in aging neuroscience. 2015;7:48.

[18] Young J, Modat M, Cardoso MJ, Mendelson A, Cash D, Ourselin S, et al. Accurate multimodal probabilistic prediction of conversion to Alzheimer's disease in patients with mild cognitive impairment. NeuroImage: Clinical. 2013;2:735-45.

[19] Moradi E, Pepe A, Gaser C, Huttunen H, Tohka J, Initiative AsDN. Machine learning framework for early MRI-based Alzheimer's conversion prediction in MCI subjects. NeuroImage. 2015;104:398-412.

[20] Landau SM, Fero A, Baker SL, Koeppe R, Mintun M, Chen K, et al. Measurement of Longitudinal β-Amyloid Change with 18F-Florbetapir PET and Standardized Uptake Value Ratios. Journal of Nuclear Medicine. 2015;56:567-74.

[21] Wang Y, Necus J, Kaiser M, Mota B. Universality in human cortical folding in health and disease. Proceedings of the National Academy of Sciences. 2016;113:12820-5.

[22] Desikan RS, Ségonne F, Fischl B, Quinn BT, Dickerson BC, Blacker D, et al. An automated labeling system for subdividing the human cerebral cortex on MRI scans into gyral based regions of interest. NeuroImage. 2006;31:968-80.

[23] Prescott JW, Guidon A, Doraiswamy PM, Roy Choudhury K, Liu C, Petrella JR, et al. The Alzheimer structural connectome: changes in cortical network topology with increased amyloid plaque burden. Radiology. 2014;273:175-84.

[24] Jenkinson M, Beckmann CF, Behrens TE, Woolrich MW, Smith SM. Fsl. NeuroImage. 2012;62:782-90.

[25] Andersson JL, Sotiropoulos SN. An integrated approach to correction for off-resonance effects and subject movement in diffusion MR imaging. NeuroImage. 2016;125:1063-78.

[26] Smith SM. Fast robust automated brain extraction. Human brain mapping. 2002;17:143-55.

[27] Mori S, Crain BJ, Chacko VP, Van Zijl PC. Three‐dimensional tracking of axonal projections in the brain by magnetic resonance imaging. Annals of Neurology: Official Journal of the American Neurological Association and the Child Neurology Society. 1999;45:265-9.

[28] Jenkinson M, Bannister P, Brady M, Smith S. Improved optimization for the robust and accurate linear registration and motion correction of brain images. NeuroImage. 2002;17:825-41.

[29] Ashburner J. VBM tutorial. Tech repWellcome Trust Centre for Neuroimaging, London, UK. 2010.

[30] Cope TE, Rittman T, Borchert RJ, Jones PS, Vatansever D, Allinson K, et al. Tau burden and the functional connectome in Alzheimer’s disease and progressive supranuclear palsy. Brain : a journal of neurology. 2018;141:550-67.
